# A new method to determine cortical bone thickness in CT images using a hybrid approach of parametric profile representation and local adaptive thresholds: Accuracy results

**DOI:** 10.1371/journal.pone.0187097

**Published:** 2017-11-06

**Authors:** Oleg Museyko, Bastian Gerner, Klaus Engelke

**Affiliations:** Institute of Medical Physics, University of Erlangen-Nuremberg, Henkestr. 91, Erlangen, Germany; Georgia Regents University, UNITED STATES

## Abstract

**Motivation:**

Cortical bone is an important contributor to bone strength and is pivotal to understand the etiology of osteoporotic fractures and the specific mechanisms of antiosteoporotic treatment regimen. 3D computed tomography (CT) can be used to measure cortical thickness, density, and mass in the proximal femur, lumbar vertebrae, and distal forearm. However, the spatial resolution of clinical whole body CT scanners is limited by radiation exposure; partial volume artefacts severely impair the accurate assessment of cortical parameters, in particular in locations where the cortex is thin such as in the lumbar vertebral bodies or in the femoral neck.

**Method:**

Model-based deconvolution approaches recover the cortical thickness by numerically deconvolving the image along 1D profiles using an estimated scanner point spread function (PSF) and a hypothesized uniform cortical bone mineral density (reference density). In this work we provide a new essentially analytical unique solution to the model-based cortex recovery problem using few characteristics of the measured profile and thus eliminate the non-linear optimization step for deconvolution. Also, the proposed approach allows to get rid of the PSF in the model and reduces sensitivity to errors in the reference density. Additionally, run-time and memory effective computation of cortical thickness was achieved with the help of a lookup table.

**Results:**

The method accuracy and robustness was validated and compared to that of a deconvolution approach recently proposed for cortical bone and of the 50% relative threshold technique: in a simulated environment with noise and various error levels in the reference density and using CT acquisitions of the European Forearm Phantom (EFP II), a modification of a widely used anthropomorphic standard of cortical and trabecular bone compartments that was scanned with various scan protocols.

**Conclusion:**

Results of simulations and of phantom data analysis verified the following properties of the new method: 1) Robustness against errors in the reference density. 2) Excellent accuracy on ground truth data with various noise levels. 3) Very fast computation using a lookup table.

## Introduction

Low bone mineral density (BMD) is an important risk factor for osteoporotic fracture. Cortical BMD and perhaps cortical thickness are independent predictors of bone strength but there is still no agreement on the relative contributions of trabecular and cortical BMD [1, 2]. Quantitative CT (QCT) is the method of choice to separately assess cortical and trabecular compartments in proximal femur and lumbar spine, primary sites for diagnosis of osteoporosis. However, due to limited spatial resolution of the imaging process the measurement of cortical parameters is often associated with larger accuracy errors, in particular in locations such as the femoral neck. Here the cortex is often thinner than 1 mm compared to a typical voxel size of 0.3 × 0.3 × 1 mm^3^ for clinical QCT. Limited spatial resolution causes partial volume artefacts, which prevent the accurate segmentation of cortical bone and as a consequence the accurate determination of cortical BMD, BMC, and thickness.

Global thresholds and the so called local adaptive threshold or 50%-method (LAT50) have been widely used for cortical bone segmentation. Global threshold based techniques in general can only provide an accurate thickness measurement if the threshold is specifically selected for a given cortical thickness [3, 4], which is impossible if the thickness varies locally such as in human bone. The LAT50-method is accurate for thicker cortices but significantly overestimates thickness below 1–2 mm because here the main assumption of the method, $\int_{\text{outside}\;\text{true}\;\text{cortex}}\;\text{PSF}\left(t\right)\;dt\approx 0$, is no longer valid [5]. The overestimation of cortical thickness or volume results in an underestimation of cortical BMD. Consequently, cortical bone mineral content, the product of density and volume, shows better accuracy for thin cortices [6–8].

A novel approach based on a deconvolution method (DM) has recently being proposed in [7, 9, 10]. With this technique, the imaging process is modeled as a convolution of the bone image with the scanner point spread function (PSF). Accurate cortical bone thickness values can be obtained by numerically deconvolving the given image. Typically, a 1D deconvolution along profiles perpendicular to the cortical bone surface is implemented to determine local cortical thickness. However, in contrast to LAT50 this approach requires knowledge of the bonewide constant true cortical density, the so called reference BMD (BMD_ref_), which also allows to regard the cortex as a box shape in the deconvolution process. Second, the scanner PSF is assumed to be a Gaussian and third, densities of trabecular and soft tissue located on either sides of the bone are assumed to be constant for a given profile. Recent studies demonstrated an improved accuracy of DM in measuring cortical thickness but DM is an optimization process which is not guaranteed to converge to a global optimum and results are sensitive to errors in the model parameters such as BMD_ref_ [7].

Here, we propose an alternative to the model-based deconvolution termed MPA (Model-based Profile Analysis) which allows to estimate cortical thickness *t*_*c*_ as:

$$\begin{array}{c} t_{c}=\frac{{\text{BMC}}_{\text{cort}}}{{\text{BMD}}_{\text{ref}}}, \end{array}$$ (1)

where in analogy to [9] BMD_ref_ is known and BMC_cort_ is the true cortical bone mineral content. The main idea is to estimate BMC_cort_ analytically from 1D profiles as measured between two 50%-points determined by the LAT50-method. Initial results of our new approach have been presented earlier in [11]. Here, we extended this work by: 1) a sensitivity analysis of the new algorithm with respect to measurement errors, 2) additional phantom based validations, 3) derivation of all mathematical results, and 4) by important implementation details. The ultimate goals of this contribution are:

1. To obtain an analytical solution for cortical thickness estimation which allows for a comprehensive analysis of the problem.
2. To reduce the number of model parameters used in the DM technique.
3. To combine MPA with LAT50 in a hybrid approach, termed HMPA (Hybrid Model-based Profile Analysis) to further reduce cortical thickness estimation errors caused by the error in BMD_ref_.

## A new model-based method to assess cortical thickness

### Materials and methods

The image acquisition process can be seen as the process of linear transformation: the true signal (object) is convolved with the scanner PSF resulting in a “blurred” output image. In CT, the in-plane PSF is mainly defined by the convolution kernel used for reconstruction of 3D volume from the raw data of acquired projections and by the X-ray source: its energy, focus, aperture, and filter. Shape of soft reconstruction kernel functions is close to the Gaussian curve so that high frequency signal is suppressed: both contrast of edges and noise are reduced. Sharp kernels resemble *sinc* function and preserve more details but also noise. Additionally, across-plane PSF and z-interpolation for spiral CT affect the output [6]. Nevertheless, the overall transformation seems to resemble the convolution with a Gaussian in all cases.

We base our analysis of the imaged cortex on *profile* measurements: lines are densily placed across the cortex and (approximately) orthogonal to the outer cortical surface. The intensity measurements along each profile are modeled as a convolution of the true cortex structure under the profile with a 1D scanner overall PSF along the profile direction, which is therefore dependent on the profile orientation. As in [5] and [10], we consider this PSF as a Gaussian. Similar to [7, 9, 10], we assume that the cortical BMD, BMD_ref_, is constant throughout the whole bone.

**Fig 1:**
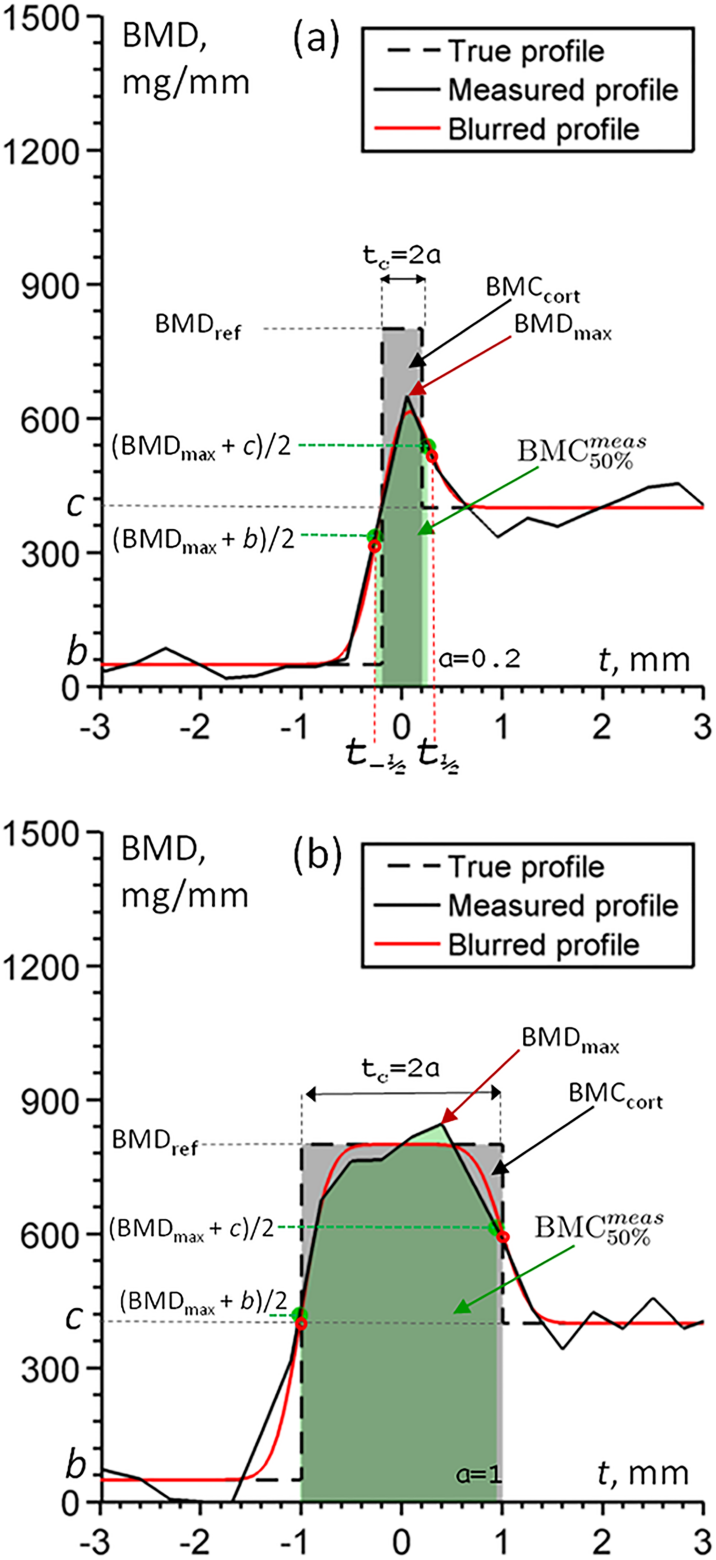
Explanation of profiles. The true profile is a piecewise constant cortical function (“step” or “box”), an ideal analytic profile (“blurred”) is a result of convolution of a Gaussian (scanner PSF) and the true profile, the measured profile is the noisy “blurred” one. Note how the position of the maximum point of the “blurred” profile depends on the thickness of the cortex (the thicker cortex, the higher BMD_max_) and the difference in *b* (soft-tissue BMD) and *c* (trabecular BMD): if *c* > *b*, then maximum is shifted towards *c*. (a) “Thin” cortex profile; (b) “Thick” cortex profile.

In the following, we will put the center of the cortex to the origin of the profile axis so that the limits of the “real” cortical bone are −*a* and *a* (*a* is unknown). The profile of the “real” cortex as a function *h*(*t*) of the position *t* along the profile axis is a box function with the height of the box BMD_ref_ as shown in Fig 1. It is surrounded by two box functions with half-infinite support on both sides of the cortex representing soft tissue density *ST*(*t*) with the height *b* and trabecular bone compartment *Tr*(*t*) with the box height (BMD) *c*. Without the loss of generality, we will always assume that “soft tissue” is on the left side of the cortex and “trabecular bone” on the right and *c* ≥ *b*. All density values in the pure 1D setting can be considered line densities (say, in mg/mm), for profiles in a 3D setting the units and interpretation of bone density and mass will be redefined.

According to the modeled imaging process, the ideal measured profile is the result of convolution of the cortex function with the imaging PSF equal to $G\left(t\right)=\frac{1}{\sqrt{2\pi }\sigma }\exp^{-\frac{t^{2}}{2\sigma^{2}}}$ (*σ* is unknown):

$$\begin{array}{c} P(t)=(G*(h+Tr+ST))(t). \end{array}$$

Since the cortex is represented as a piecewise constant function, one obtains for the model profile using the error function $\text{erf}\left(x\right)=\frac{2}{\sqrt{\pi }}\int_{0}^{x}\exp^{-t^{2}}dt$:

$$\begin{array}{c} \begin{array}{cc} P\left(t\right)=\left(G*\left(h+Tr+ST\right)\right)\left(t\right)=\frac{{\text{BMD}}_{\text{ref}}}{2} & \left[\text{erf}(\frac{t+a}{\sqrt{2}\sigma })-\text{erf}(\frac{t-a}{\sqrt{2}\sigma })\right]\\ +\frac{c}{2} & \left[1+\text{erf}(\frac{t-a}{\sqrt{2}\sigma })\right]\\ +\frac{b}{2} & [1-\text{erf}(\frac{t+a}{\sqrt{2}\sigma })]. \end{array} \end{array}$$ (2)

The detailed derivation of (2) can be found in S1 Appendix. The “blurred profile” *P*(*t*), as result of the convolution, is drawn with a solid red line for two sample cortices in Fig 1.

The problem of cortical thickness estimation is an inverse one: given *P*(*t*) find *a*. The strategy we propose for this purpose is to find few profile parameters which can be both estimated analytically from the profile formula (2) and measured in the real image profile. The comparison of these theoretical and measured values will result in a system of equations for the cortical half-thickness *a*. The selected parameters shall be robust: *a* shall not be too sensitive to the errors made in their measurements. The LAT50-method, although not accurate for the thin cortex, is known to be robust [7]. Thus, the LAT50-parameters [12] will be *measured* (and not analytically estimated) at the profile, constituting the input of our algorithm:

- *c* (and its normalized value $R=\frac{c}{{\text{BMD}}_{\text{ref}}}$): mean density of the trabecular compartment of the profile;
- *b* (normalized counterpart is $S=\frac{b}{{\text{BMD}}_{\text{ref}}}$): mean density of the soft tissue compartment of the profile;
- BMD_max_ (normalized: $T=\frac{{\text{BMD}}_{\text{max}}}{{\text{BMD}}_{\text{ref}}}$): maximal density value along the profile.

These values define the 50%-points $t_{-\frac{1}{2}}$ and $t_{\frac{1}{2}}$ which will be *analytically estimated* (measured 50%-points will be used only to obtaim the cortex BMC below, although one could use them directly in an alternative algorithm, see Discussion and conclusion):

$$\begin{array}{c} P\left(t_{-\frac{1}{2}}\right)=\frac{{\text{BMD}}_{\text{max}}+b}{2},\;P\left(t_{\frac{1}{2}}\right)=\frac{{\text{BMD}}_{\text{max}}+c}{2}, \end{array}$$ (3)

see also Fig 1. Finally, BMC-value as the integral of density values between $t_{-\frac{1}{2}}$ and $t_{\frac{1}{2}}$ will be *both measured and analytically estimated*: ${\text{BMC}}_{50\%}^{meas}$ will denote the value measured in the profile according to LAT50, BMC_50%_ will designate the estimated value. For model profiles in synthetic 1D experiments this is just the measure of mass distributed along the profile. For profiles in 3D QCT it has units of mass per area, g/cm^2^, as a product of QCT density values measured in mass per volume units and of profile lenght and will be called cortical mass surface density (“surface density” for short), cf. [10].

${\text{BMC}}_{50\%}^{meas}$ is very close to the true cortical mass BMC_cort_ if the cortex is “thick” (see Fig 1b), but also for the “thin” cortex its value is less affected than the 50%-based cortical thickness, which is overestimated, since density values at cortex are decreased due to partial volume artefacts. We are going to base our method on ${\text{BMC}}_{50\%}^{meas}$ but not with the direct equation ${\text{BMC}}_{50\%}^{meas}=\int_{t_{-\frac{1}{2}}}^{t_{\frac{1}{2}}}P\left(t;a\right)\;dt$, which is too involved; instead, we will analytically estimate the ratio $K:=\frac{{\text{BMC}}_{50\%}}{{\text{BMC}}_{\text{cort}}}$. Since BMC_cort_ as an area of the cortical “box” is equal to 2*a*BMD_ref_ (this mass value is preserved for the “blurred” cortex), *K* equals $\frac{{\text{BMC}}_{50\%}}{2a{\text{BMD}}_{\text{ref}}}$. $\frac{{\text{BMC}}_{50\%}}{2a}$ can be easily obtained, as we will see below, using BMD_max_, *c*, and *b*.

Finally, *K* is then used as a correction factor for ${\text{BMC}}_{50\%}^{meas}$ to get the value of BMC_cort_ as $\frac{{\text{BMC}}_{50\%}^{meas}}{K}$. Substituting this expression for BMC_cort_ into (1), we obtain the model-based cortical half-thickness:

$$\begin{array}{c} a=\frac{{\text{BMC}}_{50\%}^{meas}}{2{\text{BMD}}_{\text{ref}}K}. \end{array}$$ (4)

Summarizing, the method gives an estimation of the discrepancy between BMC_cort_ and BMC_50%_. The estimation is combined with ${\text{BMC}}_{50\%}^{meas}$ to produce the value of BMC_cort_ and, consequently, of *a*.

The sensitivity analysis with respect to the profile parameters and BMD_ref_ is in Sensitivity analysis and in Results. Alternative parameter sets are considered in Discussion and conclusion.

#### Estimation of the cortical BMC

Our purpose now is to estimate BMC_50%_ for the model profile *P*(*t*) and to compare it with BMC_cort_ = BMD_ref_ ⋅ 2*a* (true cortex BMC). The BMC for the part of the profile within any two points with coordinates *t*_1_ and *t*_2_ is the following area under the curve (AUC):

$$\begin{array}{c} \int_{t_{1}}^{t_{2}}P\left(t\right)\;dt=\int_{t_{1}}^{t_{2}}\left(G*h\right)\left(t\right)\;dt+\int_{t_{1}}^{t_{2}}\left(G*Tr\right)\left(t\right)\;dt+\int_{t_{1}}^{t_{2}}\left(G*ST\right)\left(t\right)\;dt. \end{array}$$

Let us consider each of three integrals in turn using Er to denote the primitive of erf(see S2 Appendix for its properties):

$$\begin{array}{c} \int_{t_{1}}^{t_{2}}\left(G*h\right)\left(t\right)\;dt=\frac{{\text{BMD}}_{\text{ref}}}{2}\int_{t_{1}}^{t_{2}}[\text{erf}(\frac{t+a}{\sqrt{2}\sigma })-\text{erf}(\frac{t-a}{\sqrt{2}\sigma })]\;dt \end{array}$$

(substituting $z=\frac{t+a}{\sqrt{2}\sigma }$ and $w=\frac{t-a}{\sqrt{2}\sigma }$ and using the evenness of Er)

$$\begin{array}{c} =\frac{{\text{BMD}}_{\text{ref}}\sigma }{\sqrt{2}}[\int_{\frac{t_{1}+a}{\sqrt{2}\sigma }}^{\frac{t_{2}+a}{\sqrt{2}\sigma }}\text{erf}\left(z\right)\;dz-\int_{-\frac{t_{1}-a}{\sqrt{2}\sigma }}^{\frac{t_{2}-a}{\sqrt{2}\sigma }}\text{erf}\left(w\right)\;dw]\\ =\frac{{\text{BMD}}_{\text{ref}}\sigma }{\sqrt{2}}[\text{Er}(\frac{t_{2}+a}{\sqrt{2}\sigma })-\text{Er}(\frac{t_{1}+a}{\sqrt{2}\sigma })-\text{Er}(\frac{t_{2}-a}{\sqrt{2}\sigma })+\text{Er}(\frac{-t_{1}+a}{\sqrt{2}\sigma })]. \end{array}$$

Similarly for the second integral:

$$\begin{array}{cc} \int_{t_{1}}^{t_{2}}\left(G*Tr\right)\left(t\right)\;dt & =\frac{c}{2}\int_{t_{1}}^{t_{2}}[1+\text{erf}(\frac{t-a}{\sqrt{2}\sigma })]\;dt\\ & =\frac{c}{2}\left(-t_{1}+t_{2}\right)+\frac{c\sigma }{\sqrt{2}}\int_{\frac{t_{1}-a}{\sqrt{2}\sigma }}^{\frac{t_{2}-a}{\sqrt{2}\sigma }}\text{erf}\left(z\right)\;dz\\ & =\frac{c\sigma }{\sqrt{2}}[\frac{-t_{1}+t_{2}}{\sqrt{2}\sigma }+\text{Er}(\frac{t_{2}-a}{\sqrt{2}\sigma })-\text{Er}(\frac{-t_{1}+a}{\sqrt{2}\sigma })]. \end{array}$$

Finally, the “soft tissue” integral is equal to:

$$\begin{array}{c} \frac{b}{2}\int_{t_{1}}^{t_{2}}[1-\text{erf}(\frac{t+a}{\sqrt{2}\sigma })]\;dt=\frac{b\sigma }{\sqrt{2}}[\frac{-t_{1}+t_{2}}{\sqrt{2}\sigma }-\text{Er}(\frac{t_{2}+a}{\sqrt{2}\sigma })+\text{Er}(\frac{t_{1}+a}{\sqrt{2}\sigma })]. \end{array}$$

Summarizing,

$$\begin{array}{c} \begin{array}{cc} \text{AUC}(t_{1},t_{2})= & \frac{{\text{BMD}}_{\text{ref}}\sigma }{\sqrt{2}}[\text{Er}(\frac{t_{2}+a}{\sqrt{2}\sigma })-\text{Er}(\frac{t_{1}+a}{\sqrt{2}\sigma })-\text{Er}(\frac{t_{2}-a}{\sqrt{2}\sigma })+\text{Er}(\frac{-t_{1}+a}{\sqrt{2}\sigma })]\\ + & \frac{c\sigma }{\sqrt{2}}[\frac{-t_{1}+t_{2}}{\sqrt{2}\sigma }+\text{Er}(\frac{t_{2}-a}{\sqrt{2}\sigma })-\text{Er}(\frac{-t_{1}+a}{\sqrt{2}\sigma })]\\ + & \frac{b\sigma }{\sqrt{2}}[\frac{-t_{1}+t_{2}}{\sqrt{2}\sigma }-\text{Er}(\frac{t_{2}+a}{\sqrt{2}\sigma })+\text{Er}(\frac{t_{1}+a}{\sqrt{2}\sigma })]. \end{array} \end{array}$$ (5)

We are going to estimate the coordinates of the 50%-points $t_{1}=t_{-\frac{1}{2}}$ and $t_{2}=t_{\frac{1}{2}}$ and then substitute them into (5) to compute the corresponding AUC value, BMC_50%_. For this, we first find the ratio $\frac{a}{\sqrt{2}\sigma }$, which we designate as $\bar{a}$, using the equation *P*(t_max_) = BMD_max_:

$$\begin{array}{c} \begin{array}{cc} \frac{{\text{BMD}}_{\text{ref}}}{2}[\text{erf}(\bar{t_{\text{max}}}+\bar{a})-\text{erf}(\bar{t_{\text{max}}}-\bar{a})]+\frac{c}{2} & \left[1+\text{erf}(\bar{t_{\text{max}}}-\bar{a})\right]\\ +\frac{b}{2} & [1-\text{erf}(\bar{t_{\text{max}}}+\bar{a})]={\text{BMD}}_{\text{max}}, \end{array} \end{array}$$ (6)

where $\bar{t_{\text{max}}}=\frac{t_{\text{max}}}{\sqrt{2}\sigma }$. The coordinate of the profile peak point, t_max_ is obtained as an extreme point of the profile curve:

$$\begin{array}{c} \begin{array}{cc} & P_{t}^{\prime }\left(t_{\text{max}}\right)=0,\\ & \frac{\sqrt{2}}{\sqrt{\pi }\sigma }(\frac{{\text{BMD}}_{\text{ref}}-b}{2}\exp^{-{\left(\bar{t_{\text{max}}}+\bar{a}\right)}^{2}}+\frac{c-{\text{BMD}}_{\text{ref}}}{2}\exp^{-{\left(\bar{t_{\text{max}}}-\bar{a}\right)}^{2}})=0,\\ & \exp[-{\left(\bar{t_{\text{max}}}+\bar{a}\right)}^{2}+{\left(\bar{t_{\text{max}}}-\bar{a}\right)}^{2}]=\frac{{\text{BMD}}_{\text{ref}}-c}{{\text{BMD}}_{\text{ref}}-b},\\ & -4\bar{t_{\text{max}}}\bar{a}=\ln(\frac{{\text{BMD}}_{\text{ref}}-c}{{\text{BMD}}_{\text{ref}}-b}). \end{array} \end{array}$$

Denote for brevity $k=-\frac{1}{4}\ln(\frac{{\text{BMD}}_{\text{ref}}-c}{{\text{BMD}}_{\text{ref}}-b})$, and we get

$$\begin{array}{c} \bar{t_{\text{max}}}=\frac{k}{\bar{a}}. \end{array}$$ (7)

Obviously, $\bar{t_{\text{max}}}$ is zero if *c* = *b*. It is shifted towards the “trabecular part” as *c* is getting larger (Fig 1a. Upon substitution of the value of $\bar{t_{\text{max}}}$ into (6) we finally obtain an equation for $\bar{a}$ with parameters BMD_ref_, BMD_max_, *c*, and *b*:

$$\begin{array}{cc} \frac{{\text{BMD}}_{\text{ref}}}{2}[\text{erf}(\frac{k}{\bar{a}}+\bar{a})-\text{erf}(\frac{k}{\bar{a}}-\bar{a})]+\frac{c}{2}[1+\text{erf}(\frac{k}{\bar{a}}-\bar{a})] & \\ +\frac{b}{2}[1-\text{erf}(\frac{k}{\bar{a}}+\bar{a})] & ={\text{BMD}}_{\text{max}}, \end{array}$$

or, using the values of *c*, *b*, and BMD_max_ normalized by BMD_ref_:

$$\begin{array}{cc} \left(1-S\right)\text{erf}(\frac{k}{\bar{a}}+\bar{a})+\left(R-1\right)\text{erf}(\frac{k}{\bar{a}}-\bar{a})-2T+S+R & =0. \end{array}$$ (8)

The function on the left-hand side of Eq (8) is strictly monotonically increasing with respect to $\bar{a}$ and has values of opposite signs as $\bar{a}\to 0$ and $\bar{a}\to \infty$ (see S3 Appendix) and thus has one root only which can be efficiently found with the help of various one-dimensional optimization algorithms [13].

Having obtained the value of $\bar{a}$, we can estimate the coordinates of the 50%—points $t_{\pm \frac{1}{2}}$ according to their definition in (3). Here again, we adopt the overline notation $\bar{t_{\pm \frac{1}{2}}}$ for the values $t_{\pm \frac{1}{2}}$ divided by $\sqrt{2}\sigma$:

$$\begin{array}{c} \left(1-S\right)\text{erf}(\bar{t_{-\frac{1}{2}}}+\bar{a})+\left(R-1\right)\text{erf}(\bar{t_{-\frac{1}{2}}}-\bar{a})=T-R, \end{array}$$ (9)

$$\begin{array}{c} \left(1-S\right)\text{erf}(\bar{t_{\frac{1}{2}}}+\bar{a})+\left(R-1\right)\text{erf}(\bar{t_{\frac{1}{2}}}-\bar{a})=T-S. \end{array}$$ (10)

The function on the left-hand side of each of the equations is smooth and has a unique maximum, thus the respective root can be effectively found using 1D optimization if the search is restricted to the range $\left(-\infty ;\bar{t_{\text{max}}}\right)$ for $\bar{t_{-\frac{1}{2}}}$ and $\left(\bar{t_{\text{max}}};+\infty \right)$ for $\bar{t_{\frac{1}{2}}}$ (the respective root is unique then).

Now we are in the position to obtain the estimation for the ratio $\frac{{\text{BMC}}_{50\%}}{{\text{BMC}}_{\text{cort}}}=:K$. Denote $\bar{t_{1}}=\frac{t_{1}}{\sqrt{2}\sigma }=\bar{t_{-\frac{1}{2}}}$ and $\bar{t_{2}}=\frac{t_{2}}{\sqrt{2}\sigma }=\bar{t_{\frac{1}{2}}}$, then

$$\begin{array}{c} \begin{array}{cc} K=\frac{\text{AUC}(t_{1},t_{2})}{2a{\text{BMD}}_{\text{ref}}} & =\frac{1}{4\bar{a}}[\text{Er}(\bar{t_{2}}+\bar{a})-\text{Er}(\bar{t_{1}}+\bar{a})-\text{Er}(\bar{t_{2}}-\bar{a})+\text{Er}(-\bar{t_{1}}+\bar{a})]\\ & +\frac{R}{4\bar{a}}[-\bar{t_{1}}+\bar{t_{2}}+\text{Er}(\bar{t_{2}}-\bar{a})-\text{Er}(-\bar{t_{1}}+\bar{a})]\\ & +\frac{S}{4\bar{a}}[-\bar{t_{1}}+\bar{t_{2}}-\text{Er}(\bar{t_{2}}+\bar{a})+\text{Er}(\bar{t_{1}}+\bar{a})]. \end{array} \end{array}$$ (11)

#### Summary of the algorithm

Summarizing, the estimation of the cortical thickness is done in the following steps.

1. Measure LAT50-parameters of the profile: *T*, *R*, and *S*.
2. Obtain $\bar{a}$ as the root of Eq (8).
3. Obtain estimated coordinates of the 50%-points normalized by $\sqrt{2}\sigma$, $\bar{t_{-\frac{1}{2}}}$ and $\bar{t_{\frac{1}{2}}}$, solving (9), respectively (10).
4. Calculate the BMC correction factor *K* using (11).
5. Calculate the model-based cortical half-thickness *a* using (4).

Let us call this algorithm Model-based Profile Analysis, MPA.

#### Accuracy analysis using simulated data

We applied the MPA algorithm to estimate cortical thickness of simulated profiles for a range of modeled thickness values from 0.5*σ* up to 7*σ*. The MPA-results were compared with that of the LAT50-method and of the DM, deconvolution method which utilizes fitting of the whole profile [9].

First, Gaussian noise levels of 30 HU and 37 HU were added to the simulated profiles. These values were chosen to match noise measurements in a SIEMENS OSTEO calibration phantom scanned with 150 and 100 mAs at 120 kV (settings typically used in clinical QCT, [14]). In the second experimental setting, errors levels in BMD_ref_ of ±5% and ±10% were simulated.

#### Sensitivity analysis

In Estimation of the cortical BMC, analytical relations were obtained for the value of the model cortical half-thickness *a*. These relations take the form of implicit (for $\bar{a}$ and $\bar{t_{\pm \frac{1}{2}}}$) and explicit (for $\bar{t_{\text{max}}}$, *K*, and *a*) functions of the input arguments. These functions are smooth if the arguments remain within the considered range: ${\text{BMC}}_{50\%}^{meas}$, BMD_ref_, and BMD_max_ are positive; BMD_ref_ is greater than each of BMD_max_, *c*, and *b*; BMD_max_ is greater than *c* and *b*. (Hereinafter, *b* is assumed zero for simplicity, since consideration of *b* for the general case can be done in the full analogy to the parameter *c*). Thus, we can approximate the error Δ*a* in the computed value of *a* with respect to any admissible combination of errors in BMD_ref_, BMD_max_, ${\text{BMC}}_{50\%}^{meas}$, and *c* by means of a Taylor expansion. For example, using convenient notations $T=\frac{{\text{BMD}}_{\text{max}}}{{\text{BMD}}_{\text{ref}}}$ and $R=\frac{c}{{\text{BMD}}_{\text{ref}}}$:

$$\begin{array}{c} \begin{array}{cc} \Delta a & =a({\text{BMC}}_{50\%}^{meas}+\Delta {\text{BMC}}_{50\%}^{meas},T+\Delta T,R+\Delta R,{\text{BMD}}_{\text{ref}}+\Delta {\text{BMD}}_{\text{ref}})\\ & -a({\text{BMC}}_{50\%}^{meas},T,R,{\text{BMD}}_{\text{ref}})\\ & =a_{{\text{BMC}}_{50\%}^{meas}}^{\prime }\Delta {\text{BMC}}_{50\%}^{meas}+a_{T}^{\prime }\Delta T+a_{R}^{\prime }\Delta R+a_{{\text{BMD}}_{\text{ref}}}^{\prime }\Delta {\text{BMD}}_{\text{ref}}\\ & +\frac{1}{2}(a_{T^{2}}^{\prime \prime }\Delta T^{2}+a_{R^{2}}^{\prime \prime }\Delta R^{2}+a_{{{\text{BMD}}_{\text{ref}}}^{2}}^{\prime \prime }\Delta {\text{BMD}}_{\text{ref}}^{2})\\ & +a_{TR}^{\prime \prime }\Delta T\Delta R+a_{T{\text{BMD}}_{\text{ref}}}^{\prime \prime }\Delta T\Delta {\text{BMD}}_{\text{ref}}+a_{R{\text{BMD}}_{\text{ref}}}^{\prime \prime }\Delta R\Delta {\text{BMD}}_{\text{ref}}+\ldots \end{array} \end{array}$$ (12)

Here $\Delta T=\frac{\Delta {\text{BMD}}_{\text{max}}}{{\text{BMD}}_{\text{ref}}}$ and $\Delta R=\frac{\Delta c}{{\text{BMD}}_{\text{ref}}}$ with BMD_ref_ fixed. There is only one term with ${\text{BMC}}_{50\%}^{meas}$ since it is a multiplicative factor for *a*, see (4). As for the higher order terms, one should include the terms with second derivatives since the coordinate of the 50%-point $t_{\frac{1}{2}}$, which *a* depends on, see (11), is not a monotone function of *T*, *R* and BMD_ref_. For example, with *R* > 0 and BMD_ref_ fixed and *T* increasing (starting from a “small value” just above *R*), $t_{\frac{1}{2}}$ moves to the origin first, but then, at a certain “critical point” it starts moving in the opposite direction. To reflect this behavior one needs the second derivative of $t_{\frac{1}{2}}$ in *T* which is a part of $a_{T^{2}}^{\prime \prime }$. This effect also translates into a similar although weaker dependence of *a* on BMD_ref_ and on *R*, for which we do not present results with the second derivatives for the sake of brevity. The terms with mixed second order derivatives are not included as well. Consequently, the Taylor series above is transformed into the following formula using (4):

$$\begin{array}{c} \begin{array}{cc} \Delta a & \approx \frac{\Delta {\text{BMC}}_{50\%}^{meas}}{2{\text{BMD}}_{\text{ref}}K}-\frac{{\text{BMC}}_{50\%}^{meas}}{2{\text{BMD}}_{\text{ref}}}(\frac{K_{T}^{\prime }}{K^{2}}\Delta T+\frac{K_{R}^{\prime }}{K^{2}}\Delta R\\ & +(\frac{1}{{\text{BMD}}_{\text{ref}}K}+\frac{K_{{\text{BMD}}_{\text{ref}}}^{\prime }}{K^{2}})\Delta {\text{BMD}}_{\text{ref}}-\frac{2{K_{T}^{\prime }}^{2}-KK_{T^{2}}^{\prime \prime }}{2K^{3}}\Delta T^{2}). \end{array} \end{array}$$ (13)

The detailed derivation of $K_{T}^{\prime }$, $K_{R}^{\prime }$, $K_{{\text{BMD}}_{\text{ref}}}^{\prime }$, and $K_{T^{2}}^{\prime \prime }$ can be found in S4 Appendix.

Graphs of the derivatives $a_{{\text{BMC}}_{50\%}^{meas}}^{\prime }$, $a_{T}^{\prime }$, $\frac{1}{2}a_{T^{2}}^{\prime \prime }$, $a_{R}^{\prime }$, and $a_{{\text{BMD}}_{\text{ref}}}^{\prime }$ for a range of parameters are considered in Results to allow for visual assessment of the magnitude of the error in *a* with respect to the measurement errors in *T*, *R*, and BMD_ref_. The quality of estimation of Δ*a* via Taylor approximation as compared to the ground truth is presented for a sample combination of the input parameters and a range of measurement errors. Finally, sensitivity to errors in *T* and *R* is compared to the dependence of the LAT50-method on these paramaters.

### Results

**Fig 2:**
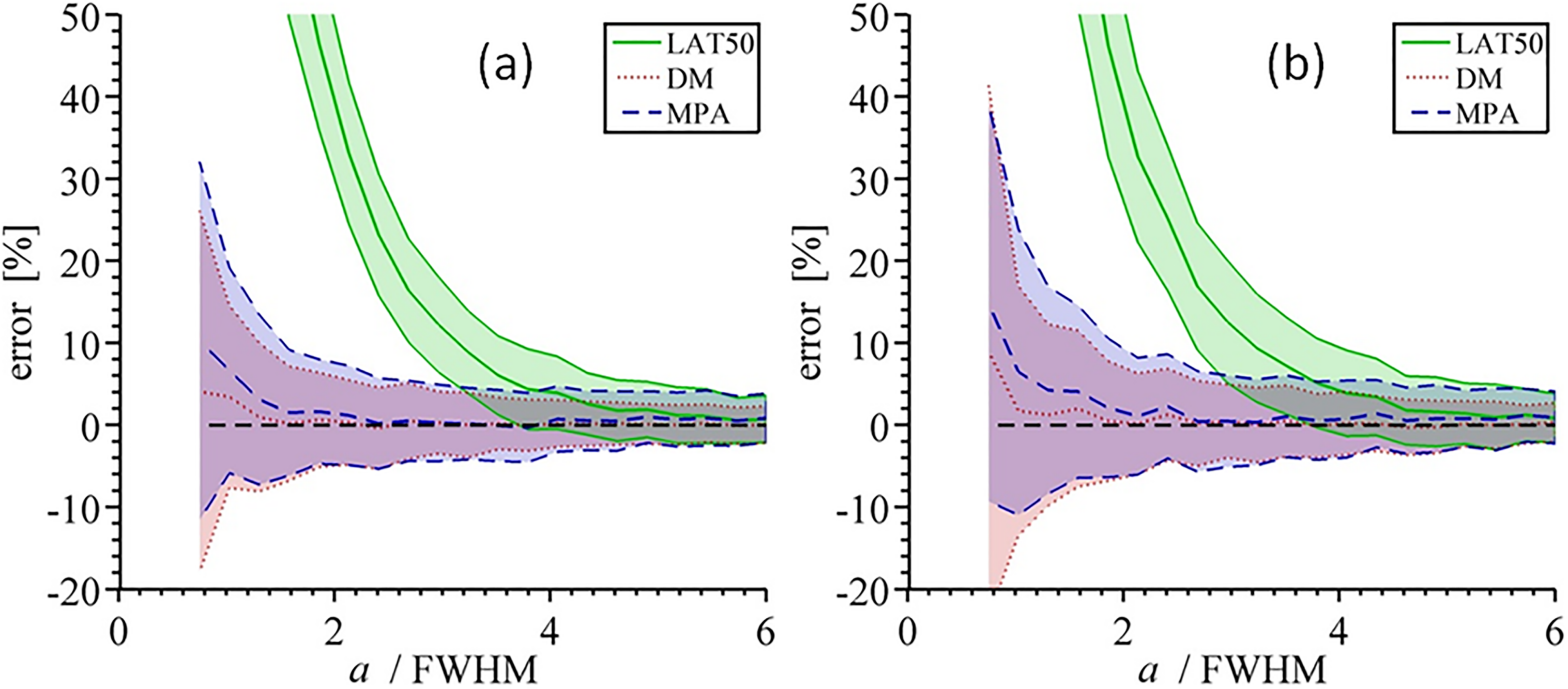
Relative error of the thickness estimation under two levels of simulated Gaussian noise. For the range of true thickness values normalized by the full width at half maximum (FWHM) of the Gaussian ($a/\text{FWHM}=a/(2\sqrt{2\ln2}\sigma )$), mean value and standard deviation of the corresponding relative error were computed after 250 simulations. These statistical parameters are shown for three methods using three different colors. True reference BMD was used (800 mg/mm), *σ* = 1.5 mm, *c* = 150, and *b* = 0 mg/mm. (a) Noise level = 30 mg/mm; (b) Noise level = 37 mg/mm.

**Fig 3:**
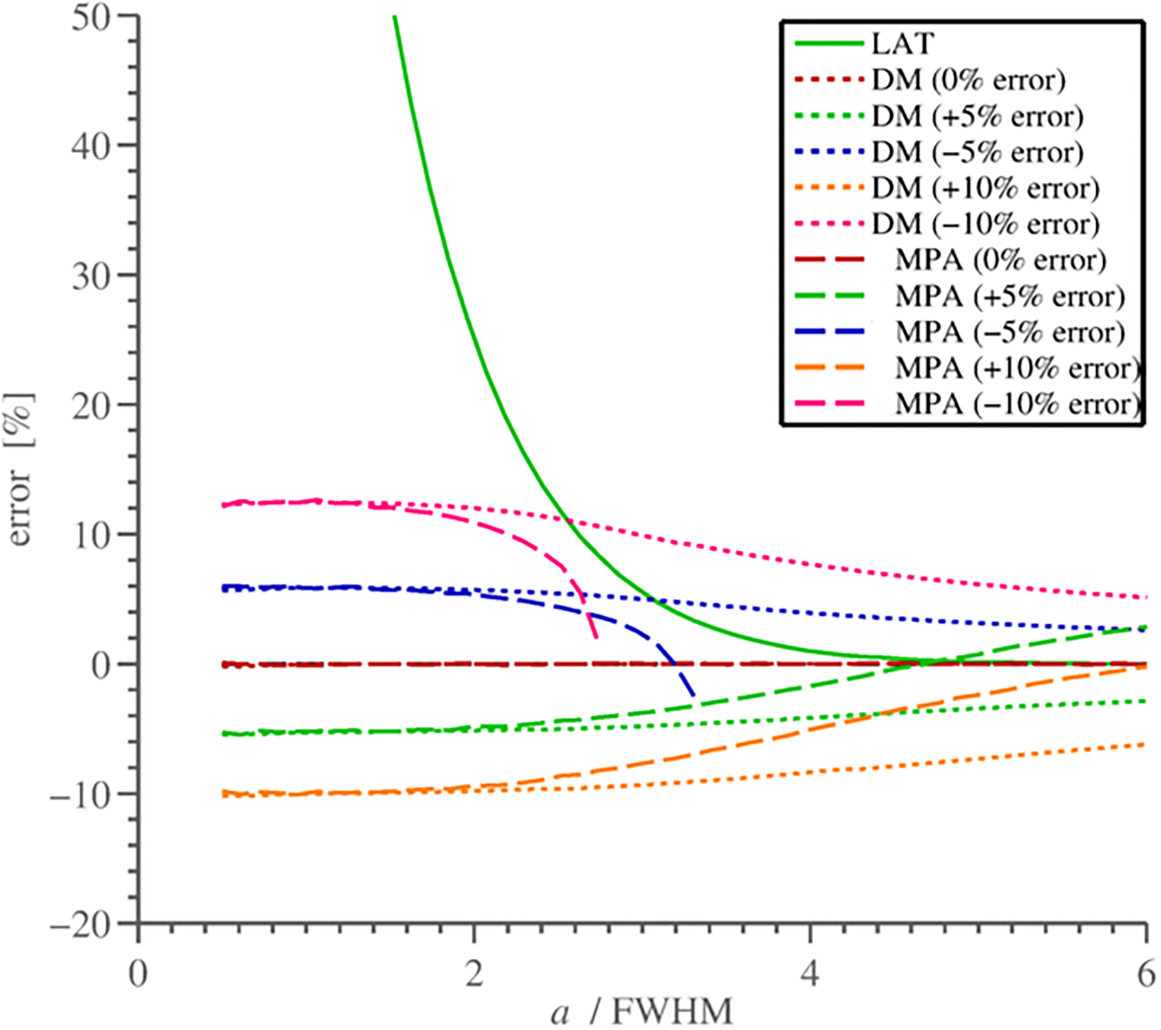
Relative thickness estimation error with respect to the error in BMD_ref_. The true level of BMD_ref_ was 800, *σ* = 1.5, and *c* = 150 mg/mm, $\text{FWHM}=2\sqrt{2\ln2}\sigma$. Note that with true BMD_ref_ both DM and MPA-methods produce no errors: the corresponding curves coincide with the x-axis.

The cortical thickness estimation by the DM, LAT50, and MPA methods under simulated noise (as described in Accuracy analysis using simulated data) are presented in Fig 2. The effect of over- and underestimation of BMD_ref_ on the performance of MPA as compared to DM and LAT50 is shown in Fig 3. One can see that error curves for MPA have a singular point when underestimated BMD_ref_ is used: it is a point where BMD_max_ approaches BMD_ref_ (i.e., *T* → 1). Since BMD_max_ cannot be greater than or equal to BMD_ref_ by definition of the profile (2), one could proceed for instance by increasing BMD_ref_ just enough to ensure the regularity of the parameters. In practice one could prefer to decrease BMD_max_ instead especially if the noise level in the image is high. We are not going to consider profile correction strategies here but postpone the question to Combination of MPA and LAT50: A hybrid method to assess cortical thickness, where a more effective method is proposed.

**Fig 4:**
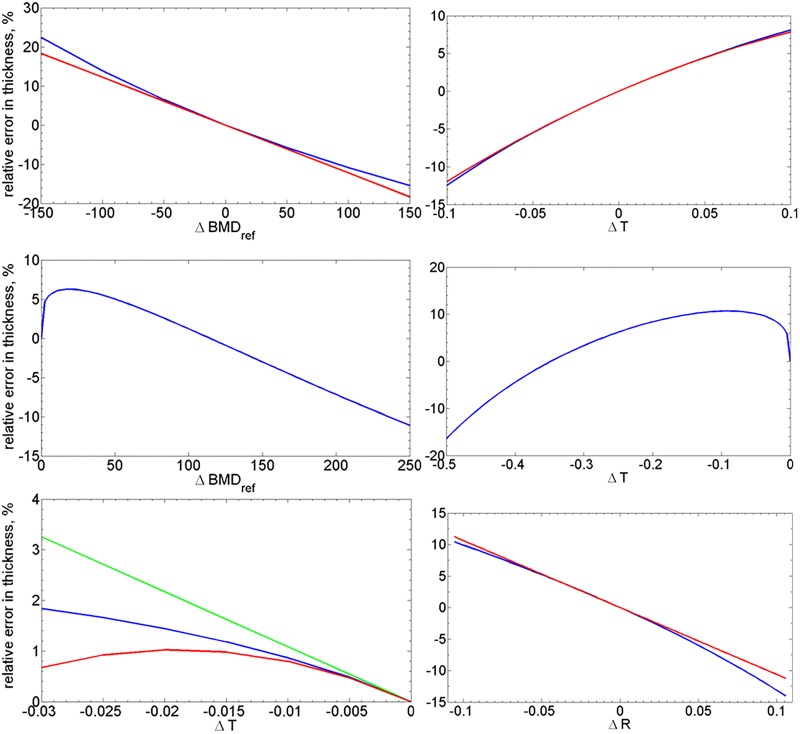
Validation of the approximate sensitivity estimation (13). The true relative errors in *a* with respect to changes in each parameter are shown in blue, corresponding approximations by Taylor expansion are in red (up to the second order term for *T* and the linear term for *R* and BMD_ref_) and green (the linear term for *T*). First row is for variation in BMD_ref_ (left) and *T* (right) at the $\bar{a}$ level of 0.5 (“thin” cortex), second row is for variation in BMD_ref_ and *T* at the thickness level $\bar{a}=3.5$ (“thick” cortex). In the third row there are results for *T* (left, $\bar{a}=1.64$) and *R* (right, $\bar{a}=0.5$). For the very thick cortex, $\bar{a}=3.5$, analytic sensitivity is not shown, as the true sensitivity becomes highly nonlinear due to vicinity of the singular point *T* = 1 (bottom left and center) and the Taylor expansion with the two first terms does not approximates it well except for a very small neighborhood of the initial value. BMD_ref_ was set to 1000 mg/mm, $R=\frac{1}{3}$, *σ* was 1.

The derivatives of *a* with respect to the independent parameters are shown as functions of BMD_ref_ in S1 Fig. The graphs of the derivatives allow for immediate estimation of the error magnitude in *a* and characterize the dependence of the magnitude on the parameters. An example computation of relative errors in the estimation of *a* using sensitivity analysis is shown in Fig 4, where also the accuracy of sensitivity analysis is validated using respective ground truth values. Note how accurately the dependence of *a* on parameters can be approximated with only linear terms if *a* is “small”, and starts exposing a non-linear behavior at higher thickness values.

In order to better understand the magnitude of the error from the sensitivity analysis, we compared sensitivity of MPA with the dependence of the 50%-thickness on the maximum and minimum values (S2 Fig and S5 Appendix). Note however that this dependence of the 50%-thickness does not take account of errors in the position of 50%-points which are caused by noise between profile maximum and minimum points.

## Combination of MPA and LAT50: A hybrid method to assess cortical thickness

### Methods: Hybrid algorithm for cortical thickness measurement

Cortical thickness estimation by MPA or DM is more accurate than that by LAT50, in particular for the “thin cortex”. However, the dependence on BMD_ref_ (and *σ* in case of DM, [9]) may outweigh the higher accuracy if the errors made in the parameter measurements are large enough. This is more likely to occur at the “thick” cortex (above 3 mm in QCT), where LAT50-estimation is rather accurate. Naturally, a hybrid method applying the model-based cortex recovery for the “thin” cortex and switching to the 50%-criterion for the “thick” cortex would be advantageous.

Let us consider the results of the MPA-algorithm as compared to that of LAT50, see Fig 3. The errors in BMD_ref_ lead to thickness overestimation, both if BMD_ref_ is set too low but also if it is too high, in the latter case the thickness is underestimated for “thin” and “midrange” cortex and is overestimated if cortex is “very thick”. On the other hand, LAT50 is independent of BMD_ref_ and shares the rest of parameters with MPA. This, together with the fact that LAT50-estimation of the cortical thickness is always larger than the true one (at least in the ideal, noiseless profile) makes the LAT50-thickness *a*_50%_ a natural upper bound for the *hybrid thickness estimation*: if

$$\begin{array}{c} a_{MPA}\geq a_{50\%} \end{array}$$ (14)

holds then *a*_50%_ is more accurate than *a*_*MPA*_. Thus, the switching point of the combined algorithm, called a hybrid model-based profile analysis (HMPA) below, is defined by (14): the lowest value between *a*_*MPA*_ and *a*_50%_ is chosen for any set of parameters.

### Implementation details

When applying the 1D profile-based cortex analysis to 3D data, a number of implementation issues should be resolved.

First, a rather complicated question of direction of the profile for the thickness measurement in 3D data has to be addressed. There are various methods for the thickness measurement, many of which go beyond the profile-based approach, for instance: 1) profile is set normal to the periosteal bone surface, 2) maximal sphere fitting [15], 3) annular approximation (cortical thickness = cortical volume/periosteal surface, see [16], e.g.), 4) Laplace equation for the point correspondence (originally used for the measurement of the brain cortex thickness [17, 18]). Note that 2)—4) require segmented cortex boundaries but are less sensitive to the “ragged” surface, 3) computes the approximate mean thickness only (no profiles needed), and 4) produces generally curved profiles. In [9], 1) was used: profiles were laid normal to the presegmented periosteal surface and the recovered cortical end points of the cortex along this profile defined the cortical thickness value.

**Fig 5:**
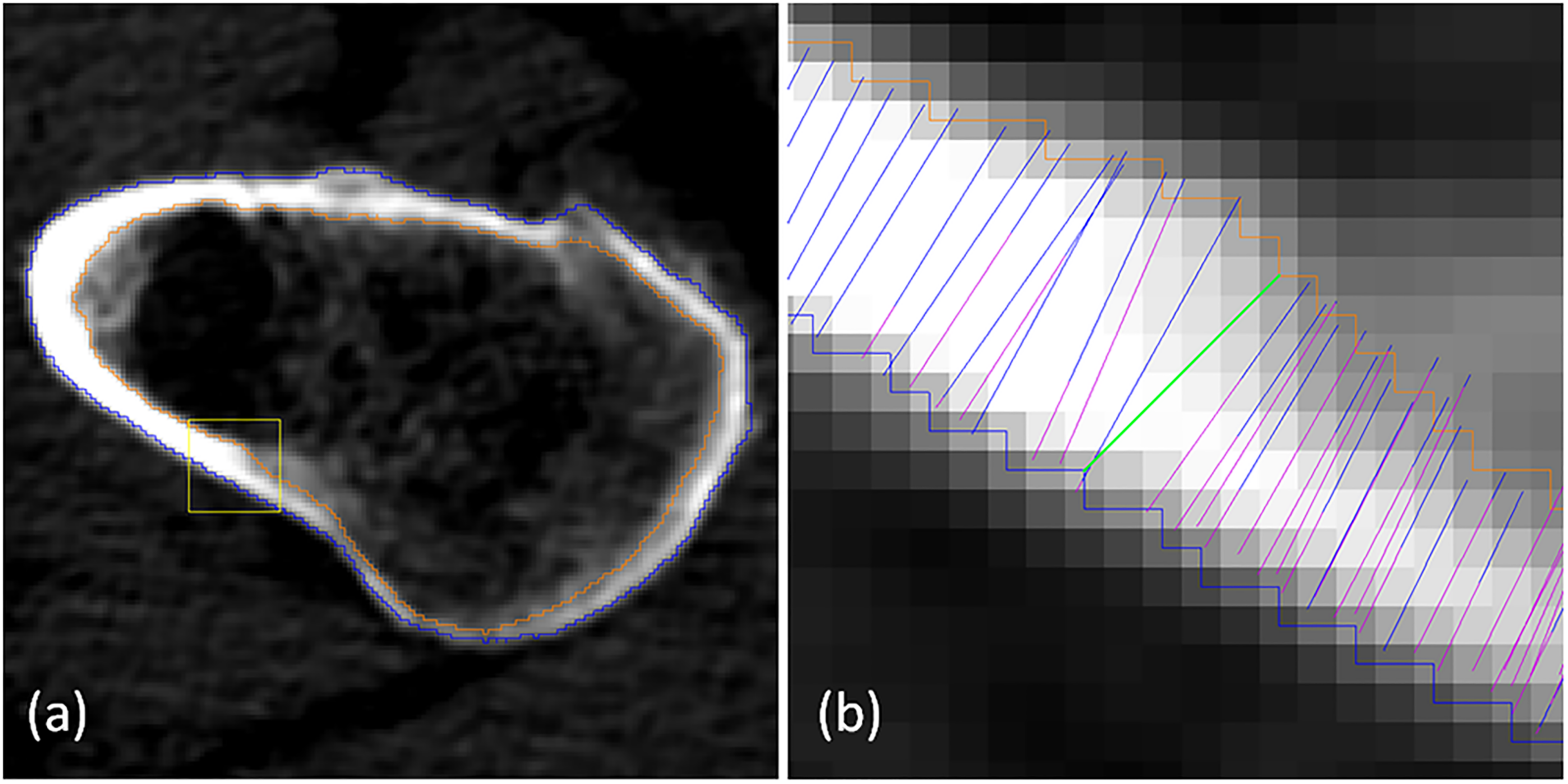
Detailed view of HMPA-segmentation. (a) An example of an axial slice with proximal femur segmentation; (b) Magnified part of the cortex with profiles: original profiles are in blue/magenta, the green line exemplifies the cortical thickness measurement at a single voxel as a voxel-to-surface distance based on segmentation. Note how direction and length of a profile are related.

However, this approach tends to *overestimate* the cortical thickness when the profile deviates from the true normal direction, see Fig 5. Therefore, when analysing the CT acquisitions of the phantom in this study, we measured the thickness in accordance to the implementation of the LAT50-method in MIAF software [12] which allows to eliminate the role of profiles with outlying normals: first, the profiles are set normal to the presegmented periosteal surface and the cortex end points are computed according the algorithm as usually, but these end points do not define the cortical thickness. Instead, all outer end points (respectively, inner points) are used to reconstruct the outer (respectively, inner) surface creating segmentation of the cortex. The cortical thickness at each point on the outer surface is defined then as the shortest distance from this point to the inner surface.

Opposite to the purely profile-based thickness estimation, this approach has bias towards the thickness underestimation (Fig 5) which is however rather limited as compared to the possible overestimation on the outlying profiles. Also, segmentation-based thickness estimation introduces an additional discretization error but has such an advantage that the segmentation mask makes the cortical volume and BMC immediately computable. The thickness values computed for each periosteal point are used to compute the mean cortical thickness in various VOIs.

Next, every profile shall be preprocessed before applying the main algorithm to reduce noise and any large deviations from the admissible profile shape prescribed by the model: thus, in [7], a profile is replaced by a result of convolution of the Gaussian with cortex model without fixed parameters (all are optimized) so that it optimally fits the measured data according to the least squares criterion. Alternatively, one could use a simple filtering such as running mean [12], which does not impose a special shape to the profile but reduces the value of BMD_max_ on average creating a bias, although mainly in “thin” profiles. Accounting for such effects may be nesessary when employing error estimations from the sensitivity analysis. The analysis of smoothing effects on the measured profile parameters is beyond the scope of this manuscript.

Finally, for the effective implementation one can use the fact that the factor *K* is a function of two parameters only (three, if one does not assume *b* being zero): *T* and *R*, allowing for run-time and size effective implementation as a lookup table with two entries. The MPA-half-thickness, *a*, is then immediately obtained by (4):

$$\begin{array}{c} a=\frac{{\text{BMC}}_{50\%}^{meas}}{2{\text{BMD}}_{\text{ref}}K\left(T,R\right)}. \end{array}$$

### HMPA validation

We tested the performance of the HMPA algorithm in simulations with errors in BMD_ref_, both over- and underestimations. To estimate the pure effect of the hybrid method, no noise was added. Same settings as for the validation of MPA were applied.

The validation with real data was based on CT scans of the European Forearm Phantom (EFP) acquired with Siemens SOMATOM Definition Flash scanner. Nine scan protocols were used, which is the number of all possible combinations of the following scan parameter values:

- Exposure: 100, 50, and 20 mAs;
- Convolution kernel: B40s (the smoothest), B50s, and B60s (the sharpest kernel).

Tube voltage of 120 kV was used in all scan protocols. In all experiments, the ground truth value of BMD_ref_ was known. In practice, one needs to obtain this parameter before starting the cortical thickness estimation algorithm.

### Results for the hybrid method

**Fig 6:**
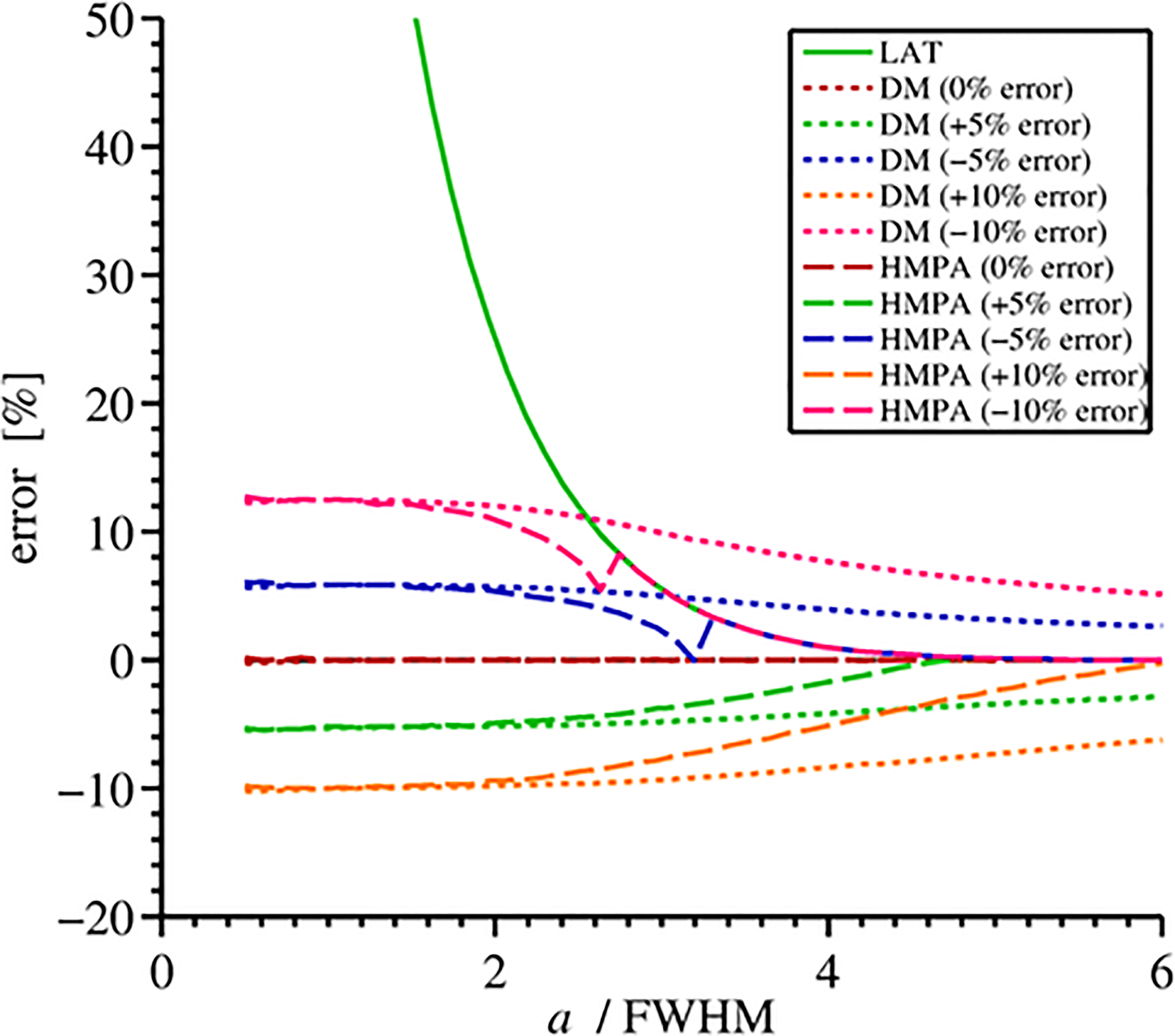
Cortical thickness estimation errors. There are shown results for DM and HMPA methods, when BMD_ref_ was measured with errors of ±5% and ±10%, and for LAT50-method which does not depend on BMD_ref_ (the solid green line). Note how the curve of the hybrid method overlaps the 50%-curve for “large” values of *a* above a certain “switching point”. (Same parameters were used as in Fig 3).

Fig 6 shows results of simulations. As compared to the MPA-method in Fig 3, the results of the hybrid method expose the effective limitation of the thickness error from above by the LAT50-curve. Note that pieces of the HMPA-curves with underestimated BMD_ref_ between singularity point (*T* = 1) and LAT50-curve are built by decreasing the value of *T* (see also Results); increasing BMD_ref_ instead would reduce the error for the corresponding thickness ranges.

**Fig 7:**
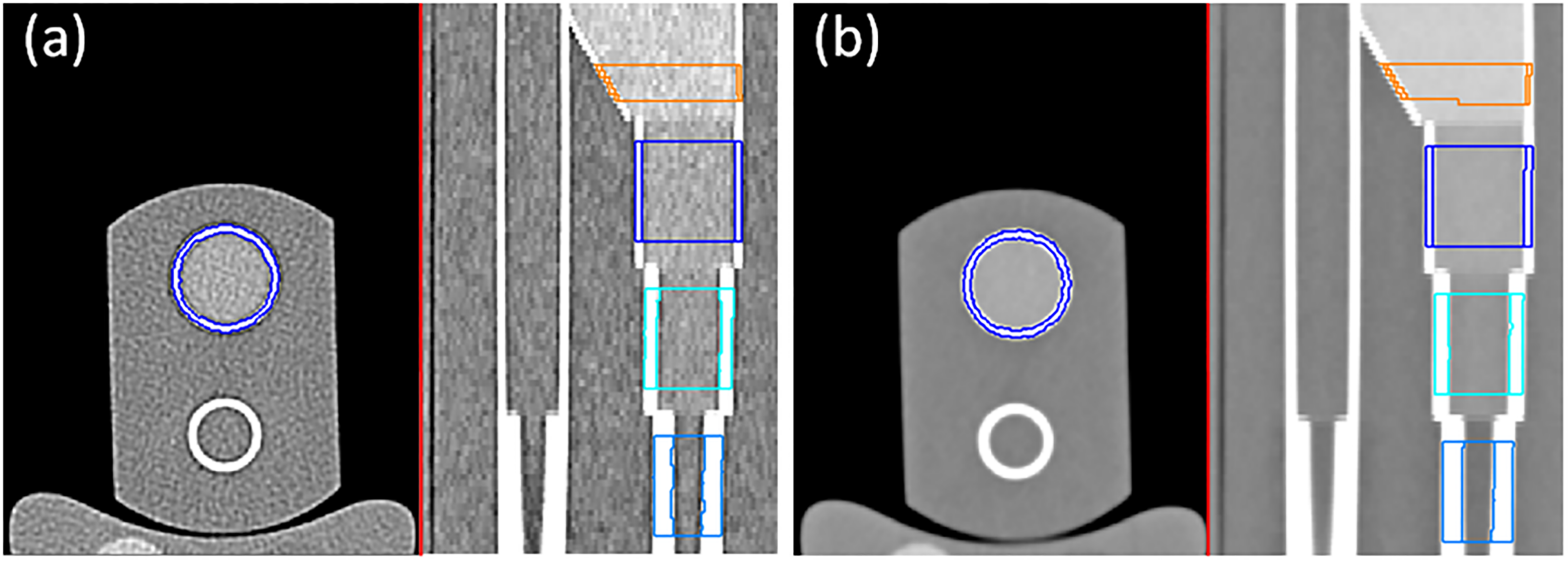
Segmentation of EFP. Two CT datasets of the European Forearm Phantom with segmentation contours. Two planar reconstructions are shown, axial and sagittal. (a) Acquired with 120 kV, 20 mAs, sharp convolution kernel B60s; (b) Acquired with 120 kV, 100 mAs, smooth convolution kernel B40s.

The results of the EFP forearm phantom segmentation are exemplified in Fig 7 for two extreme cases: the most noisy one but with highest level of image details and the most smooth one.

**Fig 8:**
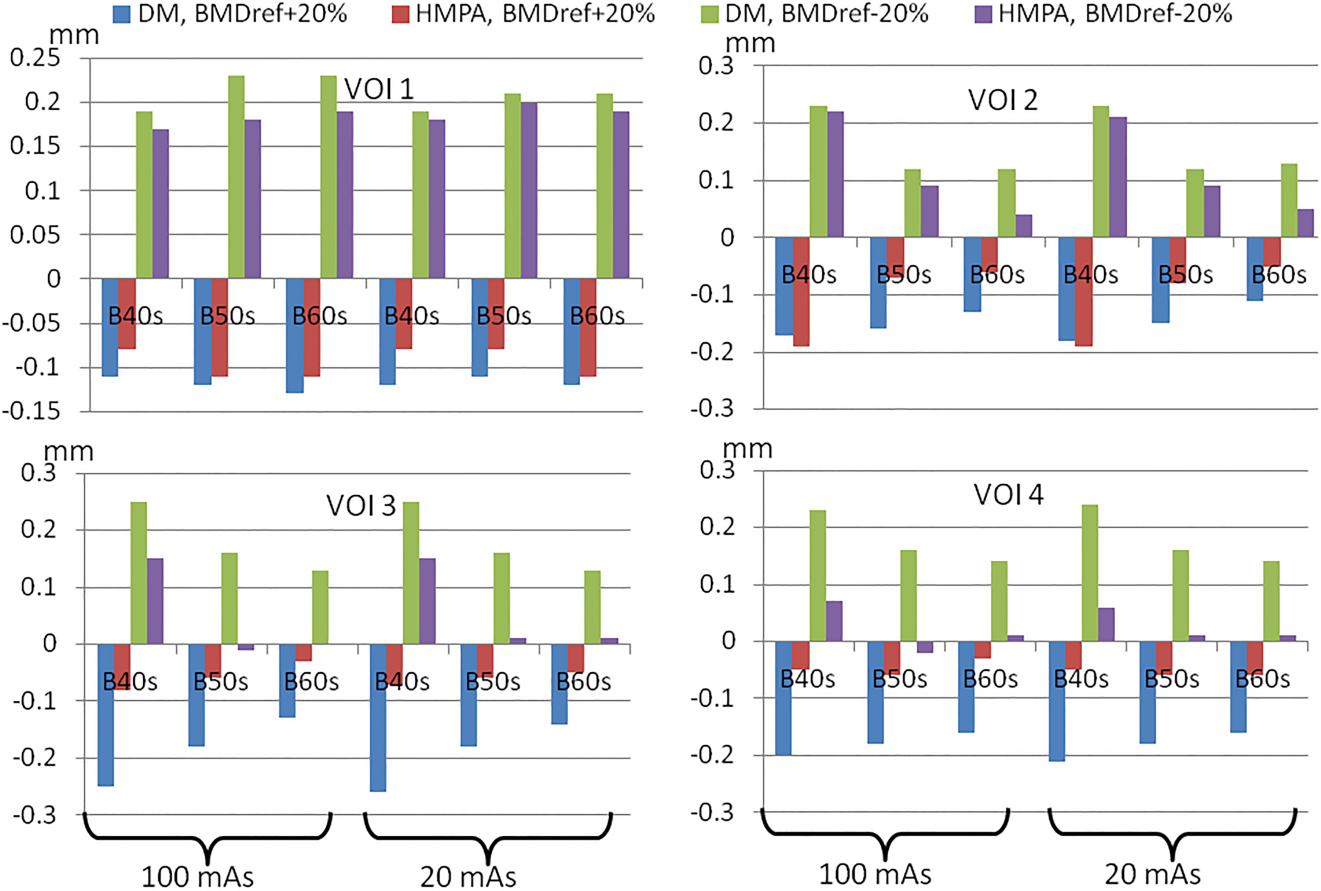
Stability of the DM and HMPA methods with respect to changes in BMD_ref_ on EFP phantom data. For each scan protocol, method, and VOI of the Table 1 the difference in 2*a* computed with the modified BMD_ref_ (±20%) and with the true BMD_ref_ is shown.

Finally, the results of cortical thickness estimation based on segmentation of EFP are shown in Table 1 and Fig 8. The following observations can be immediately made.

**Table 1:** Results of cortical thickness estimation for the EFP phantom based on segmentation with LAT50, DM, and HMPA as deviation from the ground truth. Applied BMD_ref_ was measured in the “cortex” of the shaft, the corresponding values are shown in the last row (HU).

VOI# (true 2*a*)	Method	Thickness error	100 mAs			20 mAs		
			B40s	B50s	B60s	B40s	B50s	B60s
**1** **(0.5mm)**	**LAT50**	mm	1.08	0.85	0.76	1.10	0.84	0.78
		**%**	216.5	170.3	153.0	220.3	167.9	156.2
	**DM**	mm	0.14	0.12	0.13	0.15	0.10	0.12
		**%**	27.4	23.1	25.7	29.6	19.3	23.5
	**HMPA**	mm	0.14	0.14	0.15	0.15	0.11	0.15
		**%**	27.7	28.2	30.8	29.7	22.8	29.4
**2** **(1mm)**	**LAT50**	mm	0.59	0.30	0.20	0.60	0.30	0.19
		**%**	58.7	30.2	20.5	60.5	29.5	18.9
	**DM**	mm	0.04	0.07	0.02	0.05	0.05	0.00
		**%**	4.3	7.1	1.8	5.3	5.1	0.3
	**HMPA**	mm	0.02	-0.01	-0.03	0.03	-0.01	-0.05
		**%**	1.8	-0.8	-3.2	3.1	-0.6	-4.7
**3** **(2mm)**	**LAT50**	mm	-0.14	-0.11	-0.11	-0.14	-0.13	-0.12
		**%**	-7.1	-5.5	-5.4	-7.2	-6.7	-5.8
	**DM**	mm	0.05	-0.03	-0.06	0.06	-0.06	-0.07
		**%**	2.7	-1.7	-3.1	3.0	-3.2	-3.4
	**HMPA**	mm	-0.14	-0.09	-0.08	-0.14	-0.12	-0.08
		**%**	-7.1	-4.3	-3.8	-7.2	-6.0	-4.1
**4** **(3mm)**	**LAT50**	mm	-0.12	-0.09	-0.13	-0.12	-0.12	-0.13
		**%**	-4.1	-3.2	-4.4	-4.0	-3.9	-4.4
	**DM**	mm	0.00	-0.05	-0.08	0.01	-0.07	-0.09
		**%**	0.0	-1.6	-2.5	0.4	-2.3	-3.0
	**HMPA**	mm	-0.12	-0.09	-0.11	-0.12	-0.13	-0.11
		**%**	-4.1	-2.9	-3.5	-4.0	-4.4	-3.5
	**BMD_**ref**_, HU**		1196	1218	1235	1187	1241	1246

1. When using the true BMD_ref_, DM and HMPA are quite accurate at thickness estimation except for the very thin cortex of 0.5 mm (VOI 1), where both methods overestimate *a* by about 10–15%. For the other VOIs, HMPA gives slightly but systematically lower values as compared to DM. However, it is difficult to conclude which method is more accurate since thickness computation which is based on the segmentation tends to underestimate the thickness value (shortest distance to the segmentation surface).
2. When BMD_ref_ is varied, HMPA exposes better stability of *a* than DM, which conforms with the simulation results (Fig 6).
3. The dependence on the noise level (20–100 mAs) is very low.
4. The dependence on the reconstruction kernel (B40s/B50s/B60s) is considerable.

The results for 50 mAs are not shown since they are generally very close to the range of values obtained with 20 and 100 mAs.

## Discussion and conclusion

The manuscript presents a new model-based approach (HMPA) for cortical bone segmentation in CT images. Our method shares the assumption of the known cortex density with the deconvolution method (DM) presented in [7, 9, 10] and gives theoretically equivalent results for ideal profiles. Consequently, it achieves higher accuracy at the “thin” cortex as compared to the LAT50-method but is also sensitive to errors in the main model parameter, BMD_ref_. Exactly this auxiliary parameter, BMD_ref_ distinguishes the parameter set of our method from that of the LAT50-method: BMD_max_, *b*, and *c*. Thus, on the one hand the proposed method is the parametric counterpart of DM, and on the other hand it is a direct generalization of the LAT50-method to a “thin” cortex case. The essential differences between DM and HMPA are as follows.

First, the method excludes the non-trivial optimization step and gives a solution suitable for analytical investigation. This is achieved due to the usage of few profile metrics instead of the whole curve. The use of the whole profile for fitting may be more stable, since not a single value but the whole range is used (see also Fig 2), but optimization for curve fitting is generally subject to local minima, whereas our approach provides a unique solution. Thus, the proposed method is simpler and allows for effective implementation by means of a lookup table. Computation time of the cortical thickness in the EFP with HMPA, which was less than one minute on a standard PC, was about 20–30 times shorter than that with DM. The interpretation of the accuracy as measured with the phantom (Table 1) is limited due to discretization errors (thickness is measured between voxel centers) and bias towards the lower thickness values since thickness at each point on the outer surface is the shortest distance to the inner surface. However, the sensitivity of the deconvolution method to errors BMD_ref_ is greater than that of HMPA, as illustrated in Figs 6 and 8.

Second, the proposed analytical approach eliminates the model parameter *σ*, which is defined by the scanner PSF and is essential for the deconvolution method. The *σ* may be estimated from phantom measurements, but it might be a non-trivial and/or not very accurate procedure due to the fact that the real CT-scanner PSF is not a Gaussian, especially for sharp reconstruction kernels. Also, additional parameters make the curve fitting more error prone for noisy profiles.

Third, we combine the advantages of the accurate “thin” cortex measurement provided by the model-based method (MPA) and robust and accurate estimation of the “thick” cortex provided by the LAT50-method, which is independent of errors in the model parameter BMD_ref_. The hybrid approach defines a “switch point” between the two methods. Essentially, switching to the 50%-estimation is conservative: the resulting accuracy error is generally not higher than in the case when no 50%-method was switched on at all. The dependence on the error level in BMD_ref_ is different for MPA and DM, which makes hybrid technique more advantageous for the former method leading to HMPA. Namely, LAT50-curve limits the maximum error for HMPA both when BMD_ref_ is underestimated and overestimated, whereas usage of hybrid DM-LAT50 method would limit the thickness error only when BMD_ref_ is underestimated (Fig 6).

Thanks to the ground truth phantom we could evaluate the dependence of LAT50, DM, and HMPA on noise and spatial resolution. Surprisingly, noise level did not significantly impact the results. On the contrary, the influence of the kernel was rather high and most prominent when amplified with the errors in BMD_ref_. Probably, the “smooth” kernel B40s is most similar to a Gaussian. The sharpest kernel B60s should be most different from a Gaussian, but interestingly, accuracy errors for the “middle” kernel B50s were often outside the range of that for B40s and B60s, especially for the deconvolution method. This may suggest that kernels other than Gaussian shall be considered in the model-based thickness estimation methods, too.

In the latest variant of the DM [10], a local variation of BMD_ref_ was proposed based on an empirical dependence from the estimated cortical thickness and maximal BMD value along the profile. The purpose of the introduced BMD_ref_ variation was mainly to account for cortical porosity; thus, large difference between the predicted peak BMD and the measured one was attributed to the locally decreased cortical density and used to reduce BMD_ref_. In this study, neither synthetic profiles nor the CT acquisitions of the phantom had variations in the reference BMD, i.e., the influence of this aspect was not considered when comparing the performance of the algorithms. Note however, that BMD_ref_ adjustment for porosity can make the cortical thickness artificially larger as at least in some cases the natural thickness of the porous cortex is the thickness “minus” pores, i.e., the one measured with the unadjusted BMD_ref_.

The set of the model parameters we considered is not the only one possible. For example, instead of ${\text{BMC}}_{50\%}^{meas}$ one could use the measured coordinate of a 50%-point corresponding to $t_{\frac{1}{2}}$ or $t_{-\frac{1}{2}}$: together with (9), respectively (10), one immediately obtains *σ* and then $a=\bar{a}\sqrt{2}\sigma$. However, the relative error in the measured $t_{\frac{1}{2}}$ seems to be larger than that in the corresponding BMC, as an integral value is generally more stable to local variations of the profile.

The sensitivity analysis showed that the error in the measurements causes an error of the moderate magnitude in the estimation of the cortical thickness, the sensitivity with respect to (percent) changes in BMD_ref_ was slightly higher than in other parameters. As for the the approximate sensitivity estimation based on the Taylor series, its accuracy is high for thin-to-midrange cortical thickness, and is practically useless for “thick” cortex where the maximum BMD value approaches its singular value (*T* → BMD_ref_). Since the 50%-estimation is mainly used within the hybrid method for the latter case, the sensitivity of LAT50 shall be used then.

The availability of sensitivity estimates may be used for analysis and improvement of other aspects of the model. Thus, the optimal switch point given by inequality (14) could be augmented to include an expected error in the measured BMD values at the peak of the profile (Δ*BMD*_*max*_), which can be estimated from phantom scans. However, the expected error depends on the cortical thickness: the wider the cortex, the more probable overestimation of BMD_max_ is. On the other hand, profile filtering, which is a common procedure as mentioned in Implementation details, can greatly change the measurement error. Consequently, an elaborate modeling is needed before one can say whether a more complex switch point algorithm can improve the results. Another example is the optimal discretization step of the entries of the lookup table for *K* which can be estimated based on the sensitivity analysis and selected maximum discretization error.

In patient data, the BMD_ref_ must be determined. In this study, the question of accurate BMD_ref_ estimation was not considered since it is not relevant for the ground truth data. One can use either simple methods for this, like 5% trimmed maximum value in (part of) the image, or a more complicated statistical method of [7]. We did not analyze these methods here and did not try to estimate their accuracy, since the true BMD_ref_ was known for all data we used, but we did provide the sensitivity analysis for the cortical thickness with respect to errors in BMD_ref_. Thus, one can compute the magnitude of thickness inaccuracy for any given estimation of the (average) error in BMD_ref_. Usage of real patient data obtained with various resolution levels such as standard QCT and high-resolution CT as a ground truth for an accuracy study is limited. Apart from the problem with estimation of BMD_ref_, inhomogeneous cortex structure is revealed at high resolution (porosity, adjacent trabeculae etc) which makes its appearance very different from that at the low resolution. In this view, validation with phantom segmentation is more relevant for our accuracy study which was designed to minimize unexplainable variations in the results. Essentially, the only uncontrollable source of errors in our experiments was noise, and phantom measurements were rather accurate even for very high noise levels so that influence of controllable factors could be clearly observed.

In summary, the proposed method is distinguished by the following features: 1) Robustness against errors in BMD_ref_, especially for the “thick” cortex where BMD_ref_ is not used at all. 2) Excellent accuracy on ground truth data (simulations and phantom scans) with various noise levels. 3) Very fast computation using a lookup table.

## Supporting information

S1 Appendix Profile formula (2). (TEX)

S2 Appendix Basic properties of the error function erf(t) and its primitive Er(*t*) = ∫erf(*t*)*dt*. (TEX)

S3 AppendixExistence of the unique solution $\bar{a}$ to the Eq (8).(TEX)

S4 Appendix Derivation of derivatives of *K* in BMD_ref_, *T*, *R*. (TEX)

S5 Appendix Basic results from the sensitivity analysis. (TEX)

S1 FigGraphs of the derivatives of *a* with respect to each variable as a function of BMD_ref_ at various levels of the true *a*.In the first column are the derivatives in BMD_ref_, *T*, and *T*^2^ (second derivative) at the true $\bar{a}=0.5$ (“thin” cortex). Second column: $a_{R}^{\prime }$, $a_{{\text{BMD}}_{\text{ref}}}^{\prime }$ at $\bar{a}=1.64$, and $a_{T}^{\prime }$ at $\bar{a}=1.25$. *σ* was set to 1, $R=\frac{1}{3}$.(TIF)

S2 FigDependence of the relative thickness error $\frac{\Delta a}{a}$ by LAT50 on measurment errors Δ*T* and Δ*R*.The true relative changes in *a* with respect to changes in each parameter are shown in blue, corresponding approximations by Taylor expansion are in red (up to second order terms for *T*) and green (the linear term for *T* and for *R*). First column is for variation in *T* at $\bar{a}$ levels of 0.5 and 3.5, second column is for variation in *R* at the same two thickness levels $\bar{a}$. For the “thick” cortex $\bar{a}=3.5$, only true dependence on *T* is shown (bottom left), since it is highly nonlinear at this point, which is close to the singular one (*T* = 1), and cannot be well approximated by the first two terms of the Taylor expansion.(TIF)
