# Supplementary figures and images for: A new method to determine cortical bone thickness in CT images using a hybrid approach of parametric profile representation and local adaptive thresholds: Accuracy results
Source: PLoS One. 2017 Nov 6;12(11):e0187097. doi: 10.1371/journal.pone.0187097 (PMC5673192; doi:10.1371/journal.pone.0187097)

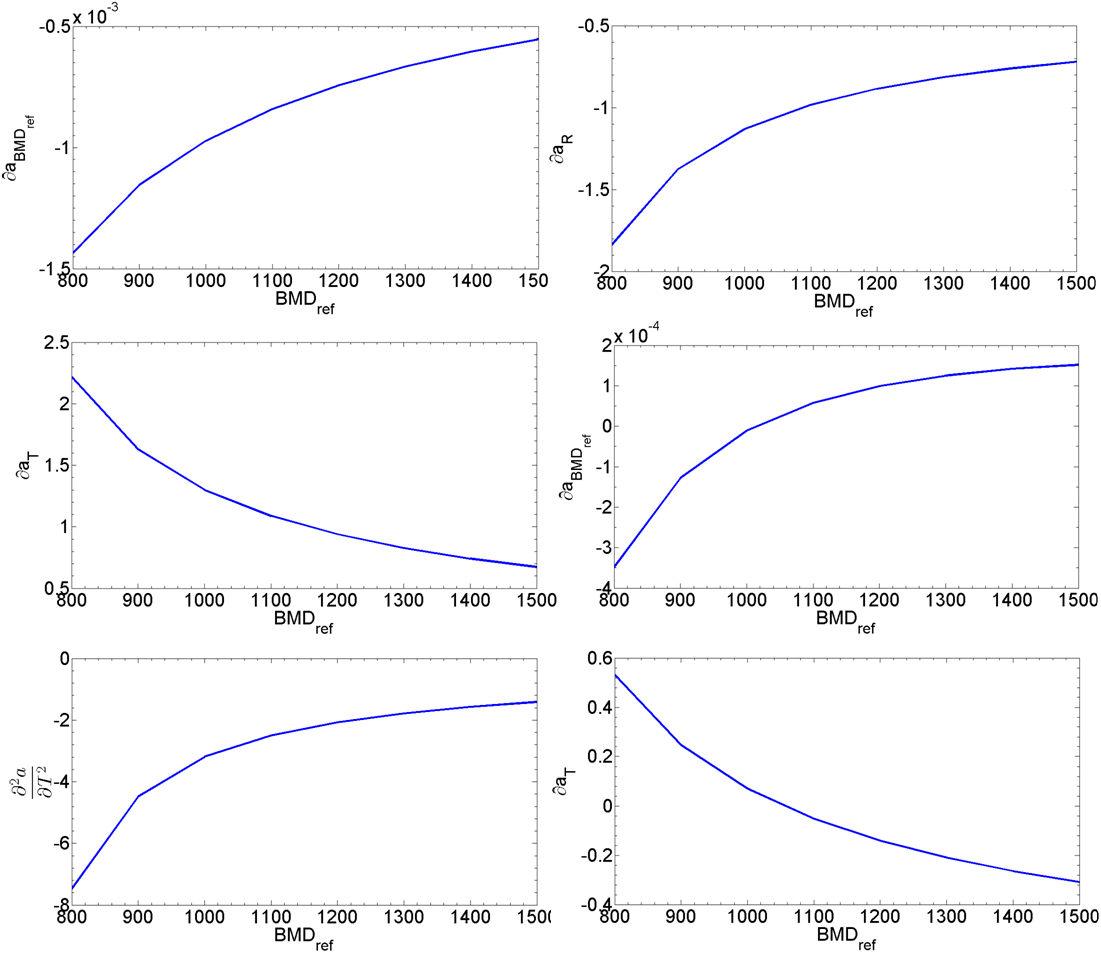

Supplement: S1 Fig — In the first column are the derivatives in BMDref, T, and T2 (second derivative) at the true a¯=0.5 (“thin” cortex). Second column: aR′, aBMDref′ at a¯=1.64, and aT′ at a¯=1.25. σ was set to 1, R=13. (TIF) [file pone.0187097.s006.tif]

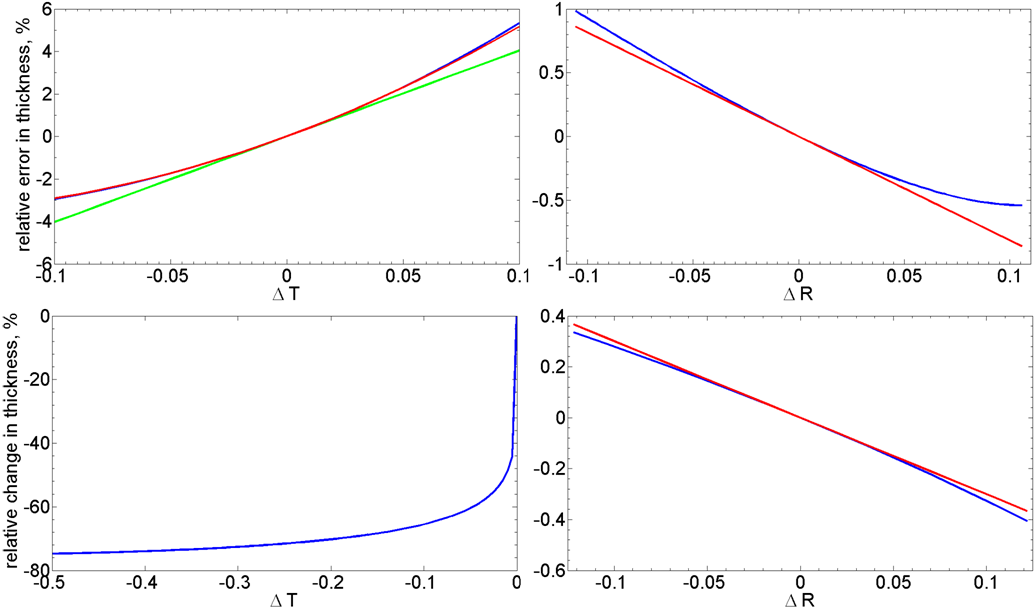

Supplement: S2 Fig — The true relative changes in a with respect to changes in each parameter are shown in blue, corresponding approximations by Taylor expansion are in red (up to second order terms for T) and green (the linear term for T and for R). First column is for variation in T at a¯ levels of 0.5 and 3.5, second column is for variation in R at the same two thickness levels a¯. For the “thick” cortex a¯=3.5, only true dependence on T is shown (bottom left), since it is highly nonlinear at this point, which is close to the singular one (T = 1), and cannot be well approximated by the first two terms of the Taylor expansion. (TIF) [file pone.0187097.s007.tif]
